# Resolution of Novel Human Papillomavirus–induced Warts after HPV Vaccination

**DOI:** 10.3201/eid2001.130999

**Published:** 2014-01

**Authors:** Steffi Silling, Ulrike Wieland, Marko Werner, Herbert Pfister, Anja Potthoff, Alexander Kreuter

**Affiliations:** University of Cologne, Cologne, Germany (S. Silling, U. Wieland, M. Werner, H. Pfister);; Ruhr University, Bochum, Germany (A. Potthoff, A. Kreuter);; Helios St. Elisabeth Hospital, Oberhausen, Germany (A. Kreuter)

**Keywords:** papillomavirus, novel human papillomavirus, HPVXS2, human papillomavirus, HPV, vaccination, quadrivalent HPV vaccine, warts, HPV vaccination, immunosuppressed, viruses

## Abstract

Human papillomavirus (HPV) XS2 was isolated from warts on an immunosuppressed patient. After HPV vaccination, the warts resolved. HPVXS2 was also found in warts and normal skin of HIV-positive patients and rarely in HIV-negative controls. Further studies should elucidate the mechanisms that lead to wart clearance.

Human papillomaviruses (HPVs), small, double-stranded DNA viruses with a circular genome of ≈8,000 bp, are assigned to different genera and species on the basis of their major capsid protein gene (L1) nucleotide sequence, which reflects their tropism (cutaneous or mucosal) and potential to induce tumors. Most HPVs belong to genera alpha (e.g., genital and wart-associated types), beta, or gamma (cutaneous types) (*1*). HPV infections are common, and the prevalence of cutaneous viral warts is 3%–5% in children (*2*). Warts, benign HPV-induced lesions, usually regress spontaneously within several months. Immunodeficiency predisposes to persistent HPV infections and the development of generalized verrucosis (*2*,*3*).

We report the remission of cutaneous warts of prolonged duration in an immunosuppressed patient after HPV vaccination. The study was performed according to the declaration of Helsinki; written informed consent was obtained from the patient.

## The Patient

In 1979, a 41-year-old, White woman received a diagnosis of B cell chronic lymphocytic leukemia and was treated with chlorambucil and prednisolone, followed by radiation therapy and splenectomy, resulting in a durable, complete remission of the leukemia. In October 2002, breast cancer was detected in the patient; the breast was surgically removed, and lymph node dissection was performed. Six cycles of chemotherapy were administered during November 2002–March 2003. In February 2010, after a 12-year history of slowly progressing cutaneous warts, the patient sought medical care for numerous, flat, erythematous warts that were coalescing into large plaques on her forearms, backs of hands, and fingers (Technical Appendix Figure). Immunophenotyping revealed a markedly decreased CD4/CD8 ratio (Table). During October 2005–December 2009, the patient received topical and ablative treatments for the warts (salicylic acid, podophyllotoxin, 5-fluoruracil cream, imiquimod 5% cream, cryosurgery, surgical curettage, electrocautery, and CO_2_ laser therapy), but clinical improvement was not sustained.

**Table:** Flow cytometric immunophenotyping results before and after human papillomavirus vaccination in a patient with splenectomy*

Cell type (reference range)	Results before vaccination†	Results after vaccination‡
CD3 cell count		
Absolute (690–2,540 cells/μL)	**2,729 cells/μL**	1,770 cells/μL
Relative (55%–84%)	**50%**	55%
CD4 cell count		
Absolute (410–1,590 cells/μL)	602 cells/μL	457 cells/μL
Relative (31%–60%)	11%	14%
CD8 cell count		
Absolute (190–1,140 cells/μL)	**2,149 cells/μL**	**1,339 cells/μL**
Relative (13%–41%)	40%	41%
CD4/CD8 ratio (0.8–2.0)	**0.28**	**0.34**

*Boldface indicates results below or above the reference range. Vaccination was started on July 19, 2010, the second dose was given 2 months later in September 2010, and the third dose was given 4 months later in January 2011. †February 2010. ‡March 2011.

Complete regression of cutaneous warts has been reported in persons after HPV vaccination (*4*,*5*); thus, we vaccinated the patient with the quadrivalent HPV (qHPV) vaccine (Gardasil, Sanofi Pasteur MSD SNC, Lyon, France), which contains L1 proteins of HPV types 6, 11, 16, and 18 as virus-like particles. Three doses were given during July 2010–January 2011. The patient’s pre- and postvaccination CD4/CD8 counts did not differ substantially (Table). In April 2011, three months after the third injection, all skin lesions had resolved (Technical Appendix Figure, panel B), and in July 2011 and March 2012, the patient was still in complete remission.

## The Study

**Figure 1:**
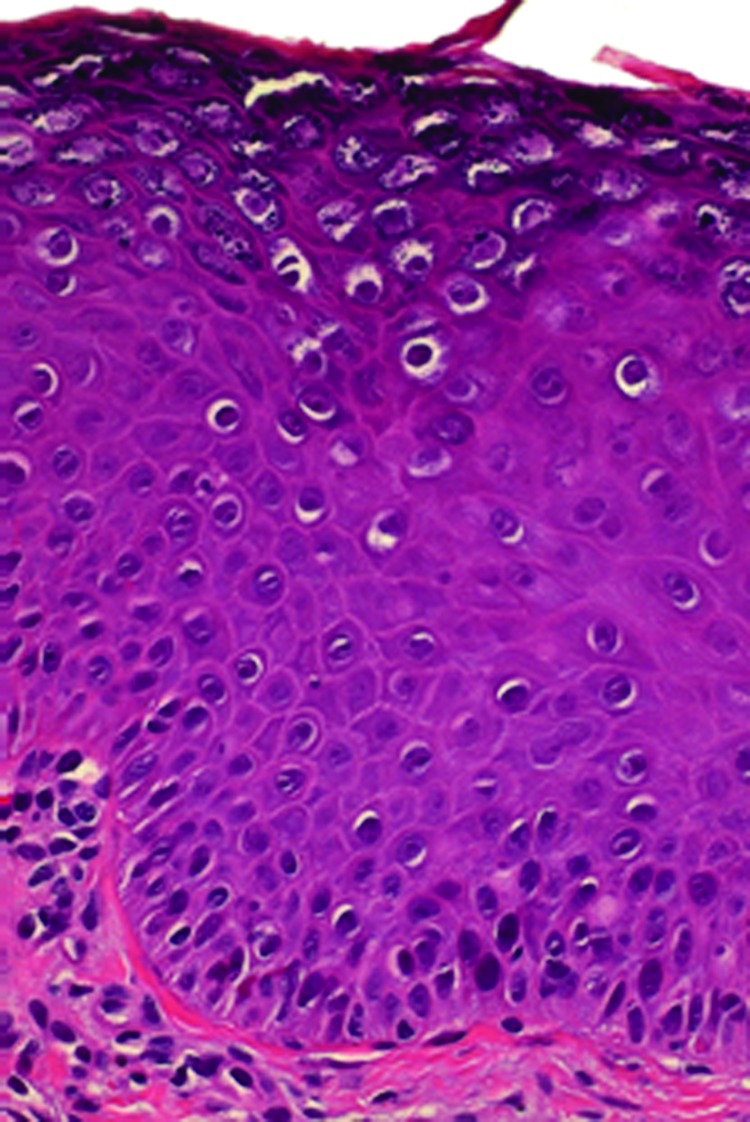
Histopathologic findings for a representative biopsy of skin lesions (erythematous warts) from a female patient before she was administered human papillomavirus vaccine. Analysis revealed features typical of benign cutaneous warts, including acanthosis, parakeratosis, and numerous koilocytes. Vacuolated granular cells show prominent keratohyalin granules, characteristic of human papillomavirus infection (hematoxylin-eosin staining; original magnification ×200).

For virologic analyses, 20 biopsy specimens from the patient’s fingers, backs of hands, and forearms and 1 specimen each from the cheek and back were available (all were collected before the patient received the first dose of qHPV vaccine). DNA extraction and HPV typing were performed as described (*6*,*7*). Histopathologic analysis revealed features typical of benign cutaneous warts, including acanthosis, parakeratosis, and numerous koilocytes (Figure 1), similar to warts caused by HPV-3 (*8*). A2/A4 PCR (*6*) was used to amplify HPV DNA from all biopsy specimens obtained before vaccination. Sequences of the PCR products were analyzed by using BLASTn (http://blast.ncbi.nlm.nih.gov) and were 100% homologous to a 261-bp fragment named HPVXS2 (*6*). Three overlapping PCR fragments covering the entire genome of HPVXS2 (7,830 bp; GenBank accession no. KC138720) were amplified by using Phusion HotStart II HF DNA Polymerase (Fermentas, St. Leon-Rot, Germany) and ligated into pJET1.2/blunt (Fermentas): fragment 1, XS2-M19fw 5′-GAATTGAGTCTTGCACCAGAGG-3′ and XhoIrev 5′-ATCTCGAGTCGCTGTCGCTTT-3′; fragment 2, XS2-M15fw 5′-GTATCTAGCACACGAGAAGTAC-3′ and XS2–6413rev 5′-ATGGTGTCCCCGACAACCC-3′; fragment 3, XS2–6258fw 5′-CACCATGTAAACAGACTGCGTC-3′ and XS2-M8rev 5′-ACCCAAATTGTTCTTTAAACTTACC-3′.

**Figure 2:**
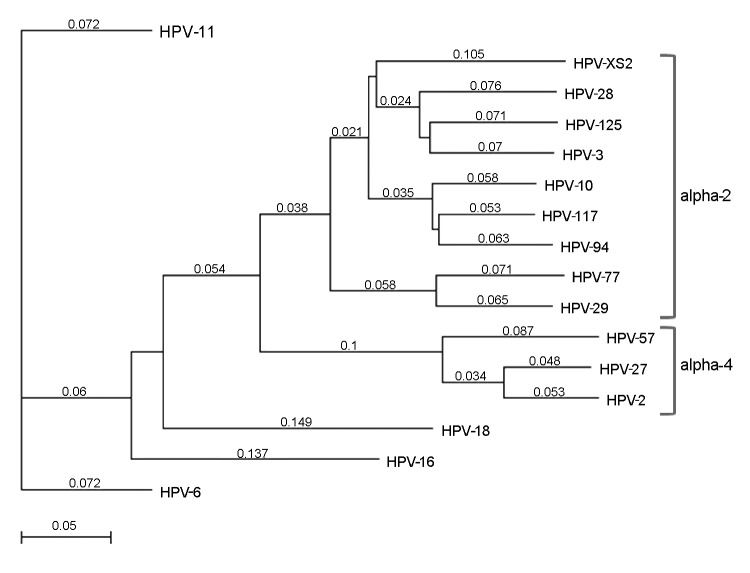
Phylogenetic tree based on selected human papillomavirus (HPV) major capsid protein gene (L1) open reading frames; the tree shows the grouping of HPVXS2. The phylogenetic analysis is based on the L1 open reading frames of all alpha-2 and alpha-4 papillomaviruses and on vaccine HPV types 6, 11, 16, and 18; the best tree was created by using the neighbor joining method with Tamura-Nei distances given. All L1 sequences were aquired from the Papillomavirus Episteme webpage (http://pave.niaid.nih.gov/#home). Scale bar indicates nucleotide substitutions per site.

MacVector software version 12.7.3 (MacVector, Inc. Cary, NC, USA) was used to determine the organization of the predicted open reading frames (ORFs) and perform phylogenetic analyses. The results showed grouping of HPVXS2 within the alpha-2 species (Figure 2), and in each case, the L1 ORF was <90% homologous to the closest relative. Thus, HPVXS2 can be considered a novel HPV type (*1*).

An HPVXS2-specific quantitative real-time PCR was established. In brief, a 20-μL reaction contained 10 μL of LightCycler 480 Probes Master (Roche, Mannheim, Germany), 0.1 μmol/L probe no. 46 (5′-ATGGCTGC-3′) of the Universal Probe Library (Roche), 0.2 μmol/L each primers XS2-L1fw 5′-CATTTGTCAGTCTGTTTGTAAATATCC-3′ and XS2-L1rev 5′-TCTGCGCAGGTAAAAGAACA-3′, and 2 μL extracted DNA (QIAamp DNA Mini Kit; QIAGEN, Hilden, Germany). Cycling conditions were 95°C for 10 min and 45 cycles at 95°C for 10 sec, 60°C for 30 sec, and 72°C for 5 sec. Virus load was expressed as HPV DNA copies per β-globin gene copy (HPV/β-globin) (*9*). In 17 of the patient’s warts, HPVXS2 loads ranged from 903 to 99,571 (median 14,534) HPV/β-globin. Virus loads were much lower in a seborrheic keratosis from her back and a benign nevus from her cheek (1.424 and 0.012 HPV/β-globin, respectively). Two skin specimens obtained 14 months after the third qHPV vaccine dose were HPVXS2-negative.

To estimate the proportion of HPVXS2-positive specimens among archived, extracted DNA, we screened 62 skin warts from 17 immunocompetent and 24 immunosuppressed patients. HPVXS2-DNA was present in warts from 3 HIV-positive women. Two warts were co-infected with HPV-57, and HPVXS2 loads were low (0.0002 and 0.034 HPV/β-globin, respectively). One wart contained HPVXS2 only (virus load 6.853 HPV/β-globin). We also screened 449 swab samples collected for a previous study; the samples were of normal forehead skin from HIV-positive men and HIV-negative male controls (*10*). HPVXS2 DNA was present in 16.2% (34/210) and 0.8% (2/239) of specimens from HIV-positive and HIV-negative men, respectively (p<0.001; χ^2^ test, 2-sided). Among the HIV-positive men, those with CD4 counts of <350 cells/μL were more likely than those with CD4 counts of >350 cells/μL to be HPVXS2-positive, but the difference was not statistically significant (25.0% vs. 13.5%, p = 0.101). Virus loads were low (0.014 and 0.023 HPV/β-globin) in the 2 HPVXS2-positive HIV-negative controls. Virus loads of >1.0 HPV/β-globin were found on normal skin of 10 of 34 HPVXS2-positive HIV-positive men (range 0.002–35.0 HPV/β-globin; median 0.107 HPV/β-globin; interquartile range 1.266 HPV/β-globin).

## Conclusions

HPVXS2 fragments were previously identified in hyperkeratotic benign papillomas, squamous cell carcinomas, and dermatitis on 3 renal transplant recipients at consecutive visits and in different parts of the body (*6*). In our study, HPVXS2 was identified in disseminated warts from a patient who had had a splenectomy and in benign skin warts from 3 HIV-positive patients. HPVXS2 DNA loads were well above 1.0 HPV/β-globin in the patient who had received a splenectomy and in an HIV-positive woman with HPVXS2 monoinfection. These virus loads are in line with those in warts induced by other HPV types (*11*). HPVXS2 was found more frequently on normal skin of HIV-positive than HIV-negative men. Thus, immunocompromised persons seem to have difficulties in clearing HPVXS2. However, virus loads remain low to moderate, and the infection in immunocompromised persons is clinically inapparent in most cases.

The patient in our study was free of warts 3 months after the last dose of qHPV vaccine, even though HPVXS2 is not closely related to the vaccine virus types (Figure 2). This observation correlates with published case reports (*4*,*5*,*12*,*13*), but it is still surprising, given that clinical improvement was not seen in 5 patients with HPV-6–positive condylomas who received the same vaccine (*14*). The qHPV vaccine was shown to induce type-specific humoral and cellular immune responses in an immunodeficient patient (*15*), but the mechanisms leading to regression of skin warts associated with heterologous HPV types have not been analyzed. One explanation could be that vaccination led to a general stimulation of the immune system, and innate immunity destroyed virus-infected cells.

We report a single observation; however, correlation does not necessarily imply causation, and a placebo effect is possible. Considering our findings, immunologic studies elucidating the mechanisms that lead to wart clearance and controlled clinical trials should be initiated.

Technical Appendix Clinical picture of the right forearm of a splenectomized patient with disseminated warts before and after human papillomavirus vaccination.
