# Supplementary material for: Resolution of Novel Human Papillomavirus–induced Warts after HPV Vaccination
Source: Emerg Infect Dis. 2014 Jan;20(1):142–5. doi: 10.3201/eid2001.130999 (PMC3884730; doi:10.3201/eid2001.130999)
Supplement: Technical Appendix — Clinical picture of the right forearm of a splenectomized patient with disseminated warts before and after human papillomavirus vaccination. [file 13-0999-Techapp-s1.pdf]

Article DOI: http://dx.doi.org/10.3201/eid2001.130999

Resolution of Novel Human Papillomavirus–induced Warts after HPV Vaccination

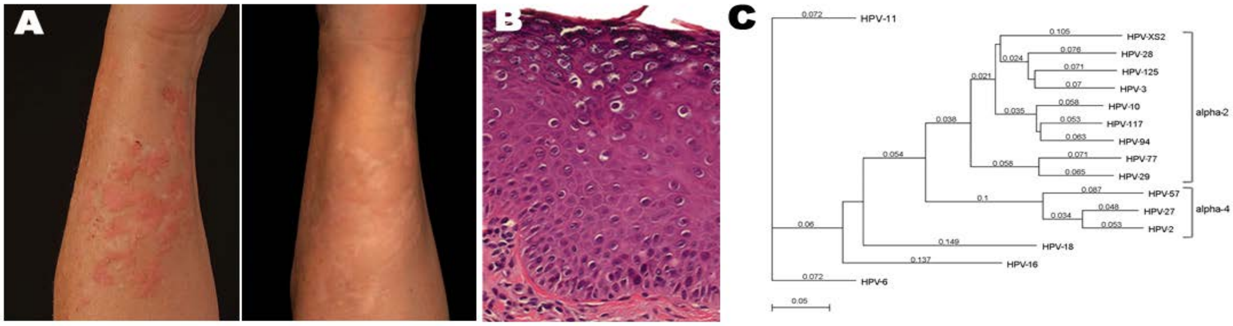

Technical Appendix

Technical Appendix Figure. Clinical picture of the right forearm of a splenectomized patient with disseminated warts before and after human papillomavirus (HPV) vaccination. A) Clinical appearance of the patient’s forearm before HPV vaccination: numerous flat erythematous papules and plaques are present (June 2010). B) Clinical appearance of the patient´s forearm 14 months after the third HPV vaccine dose (March 2012): all skin warts are cleared.
